# Oxidative stress responses to a graded maximal exercise test in older adults following explosive-type resistance training^☆^

**DOI:** 10.1016/j.redox.2013.12.004

**Published:** 2013-12-12

**Authors:** Roberta Ceci, Maria Reyes Beltran Valls, Guglielmo Duranti, Ivan Dimauro, Federico Quaranta, Monica Pittaluga, Stefania Sabatini, Paolo Caserotti, Paolo Parisi, Attilio Parisi, Daniela Caporossi

**Affiliations:** a Unit of Biology, Genetics and Biochemistry, University of Rome “Foro Italico”, Piazza Lauro De Bosis 15, 00135 Rome, Italy; b Unit of Internal Medicine, Department of Movement, Human and Health Sciences, University of Rome “Foro Italico”, Piazza Lauro De Bosis 15, 00135 Rome, Italy; c Department of Sports Science and Clinical Biomechanics, University of Southern Denmark, Campus vej 55, 5230 Odense M, Denmark

**Keywords:** Explosive-type moderate intensity resistance training (EMRT), Graded maximal exercise test (GXT), Oxidative stress, HSPs, Apoptosis, Elderly

## Abstract

We recently demonstrated that low frequency, moderate intensity, explosive-type resistance training (EMRT) is highly beneficial in elderly subjects towards muscle strength and power, with a systemic adaptive response of anti-oxidant and stress-induced markers. In the present study, we aimed to evaluate the impact of EMRT on oxidative stress biomarkers induced in old people (70–75 years) by a single bout of acute, intense exercise. Sixteen subjects randomly assigned to either a control, not exercising group (*n*=8) or a trained group performing EMRT protocol for 12-weeks (*n*=8), were submitted to a graded maximal exercise stress test (GXT) at baseline and after the 12-weeks of EMRT protocol, with blood samples collected before, immediately after, 1 and 24 h post-GXT test. Blood glutathione (GSH, GSSG, GSH/GSSG), plasma malonaldehyde (MDA), protein carbonyls and creatine kinase (CK) levels, as well as PBMCs cellular damage (Comet assay, apoptosis) and stress–protein response (Hsp70 and Hsp27 expression) were evaluated. The use of multiple biomarkers allowed us to confirm that EMRT *per se* neither affected redox homeostasis nor induced any cellular and oxidative damage. Following the GXT, the EMRT group displayed a higher GSH/GSSG ratio and a less pronounced increase in MDA, protein carbonyls and CK levels compared to control group. Moreover, we found that Hsp70 and Hsp27 proteins were induced after GXT only in EMRT group, while any significant modification within 24 h was detected in untrained group. Apoptosis rates and DNA damage did not show any significant variation in relation to EMRT and/or GXT.

In conclusion, the adherence to an EMRT protocol is able to induce a cellular adaptation allowing healthy elderly trained subjects to cope with the oxidative stress induced by an acute exercise more effectively than the aged-matched sedentary subjects.

## Highlights

• A low frequency, moderate intensity, explosive-type resistance training (EMRT) does not affect redox homeostasis at rest.
• EMRT improves the general adaptive response to oxidative stress induced by graded maximal effort.
• EMRT is effective intervention for improving the overall health of the older people.

## Graphical abstract

**Figure:**
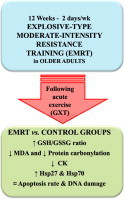

## Introduction

Aging is a multifactorial and complex process [1] characterized by a declined ability to respond to stress and to maintain cellular and systemic homeostasis [2]. Reactive oxygen species (ROS) were proposed as central components to the aging process by the Free Radical Theory of Aging [3]. In addition, they are linked to numerous pathologies like cancer, diabetes and neurological diseases [4,5] since high levels of ROS production can be damaging through oxidation to cellular components (proteins, DNA, and lipids) [6]. On the other hand, according to the hormesis theory [7], nowadays low-to-moderate levels of cellular oxidants are found to be important signals involved in muscle homeostasis like maintaining normal skeletal muscle structure and function [8].

Physical exercise is recommended for the maintenance of well-being and as a means to reduce risk for several chronic diseases. The beneficial effects of exercise might also be related to hormesis [9,10] since long-term exposure to moderate exercise is able to stimulate at the same time ROS production and antioxidant defences either in young and old people [11]. However, strenuous exercise is associated with an overproduction of ROS that can lead to increased muscle damage [12]. Scientific evidences suggest that an acute bout of exercise may cause more severe oxidative injury to muscle in aged than in young human subjects [13]. Even though physically active aged people benefit from exercise-induced adaptation in cellular antioxidant defense systems [14], age-related biochemical changes make them more susceptible to oxidative stress following intense exercise [13].

Since exercise training has been recommended to the elderly as a means to improve health, determining the appropriate exercise intensity for aged people is fundamental, in order to evaluate potential adverse effects of exercise-related ROS production. Hence, fitness trainers should be aware of the suitable type and intensity of exercise in order to achieve a correct balance between the improvement of health-related fitness capacities, exercise-related ROS production and antioxidant induction.

Oxidative stress has mainly been studied in the context of aerobic exercise while the response to resistance exercise is less defined. Among the different type of exercises recommended for older adults, several studies have incorporated explosive-type resistance training in older adults to improve muscle power, as a fundamental muscle capacity for older adults well-functioning [15]. We have recently shown that 12 weeks of a low frequency, moderate intensity, explosive-type resistance training (EMRT) may be considered an attractive exercise modality for older adults. Indeed, it concurrently improved muscle strength, power, and functional performance without detrimental effects on cardiovascular or inflammatory parameters [16].

To further analyze the systemic beneficial effect of EMRT, the current study aimed to examine the impact of EMRT protocol on cellular and biochemical oxidative stress markers at basal level and after a maximal exercise stress test (GXT) that is effective in inducing acute oxidative stress. We evaluated on blood samples: (1) redox homeostasis, through the analysis of the total glutathione (tGSH), a major hydrophilic circulating antioxidant, and reduced/oxidized glutathione ratio (GSH/GSSG); (2) oxidation target molecules monitoring both the malondialdehyde (MDA), a lipid peroxidation end-product, and the protein carbonyls (oxidized proteins) levels; (3) cellular damage by assessing plasma creatine kinase (CK) level. Peripheral blood mononuclear cells (PBMCs) were used to investigate the rate of apoptotic cell death, DNA damage, as well as the modulation of the most exercise-responsive heat shock proteins such as Hsp70 and Hsp27, effective in preventing the accumulation of oxidized proteins [17].

## Material and methods

### Study design

Recently we have conducted a controlled trial, in which participants were randomly divided into training and control group, to evaluate the effect of an *EMRT protocol* for 12 weeks on muscle strength, power and functional performance in old people (70–75 years) [16]. The study, has been expanded utilizing subjects from the trained group (*n*=8) and subjects from the control group (*n*=8) that were successfully able to complete the blood samplings that we carried out after graded maximal exercise stress test that was performed both at baseline and after the EMRT protocol experimental time. Our subset resulted to be fully representative of the original groups, being the anthropometric, physiological and functional characteristics, at both the baseline and post-EMRT training, overlapping the values of the previous study (Table 1) [16]. Physical activity level was evaluated using the Modified Baecke Questionnaire for Older Adults [18]. All subjects were fully informed of the research design and associated benefits and risks of the investigation before signing an informed consent approved by the Medical Ethics Committee of the University of Rome “Foro Italico”.

### Graded maximal exercise stress test (GXT)

The graded maximal exercise stress test was performed with a cycloergometer according to the following protocol: at first the load was fixed at 30 W for 3 min, then the exercise load was increased 10 W every minute until the achievement of 85% of maximal heart rate (HR_max_) (calculated through this formula: HR_max_=220−age in years). The test was terminated when the subject volitionally stopped exercising owing to muscular fatigue or shortness of breath, or could not maintain the designated pedaling rate for 10 consecutive seconds; then a 5-min recovery phase with a 20 W load was carried out. A continuous 12-lead ECG recording was performed and blood pressure was assessed through a mercury sphygmomanometer during the test and the following recovery.

### Blood sampling

Blood samples were collected at rest (PRE), immediately after (POST), 1 h and 24 h after GXT, at baseline (subset A) and after the experimental time 12 weeks (subset B). Blood sample collection and tests were performed early in the morning, after the night rest and overnight fasting. Test conditions were kept constant both for control and trained subjects. Blood samples were drawn from the antecubital vein while subjects remained in reclined position. Blood sampled in EDTA tubes (BD Biosciences, San Jose, CA, USA) were used for plasma collection by centrifugation of whole blood (2500 rpm×10 min at 4°C), and for PBMCs isolation (see below).

Plasma and PBMCs samples were stored at −80 °C for further analyses.

### Glutathione (GSH)

Blood GSH content was quantified spectrophotometrically at 412 nm, by a 5,50-dithiobis(2-nitrobenzoicacid) (DTNB)–GSH reductase recycling assay, accordingly to the method of Anderson [19] Briefly, 20 μl of blood samples were added to 100 μl of 10 mM HCl. Red blood cells were lysed by freezing and thawing three times; after centrifugation at 10,000*g* for 5 min at 4 °C, the supernatant solution was deproteinized with 5% 5-sulfosalicylic acid. Oxidized glutathione (GSSG) was selectively measured in samples where reduced GSH was masked by pretreatment with 2-vinylpyridine (2%).

### Protein carbonyls

Plasma protein carbonyls were determined by measuring the reactivity of carbonyl derivatives with 2,4-dinitrophenylhydrazine (DNPH) as previously described [20] with some modifications. In brief, plasma proteins (100 μl) were precipitated with 10 volumes of HCl–acetone (3:100) (v/v), then washed with HCl–acetone to remove chromophores, centrifuged (800*g*, for 20 min, +4 °C). Protein pellets were resuspended in 500 μl of PBS to which 500 μl of 10 mM DNPH (in 2 M HCl) (or 2 M HCl alone for protein blanks) was added and vortexed continuously.

After mixing, 500 μl of 30% TCA was added to each tube, placed on ice for 10 min and then centrifuged (800*g*, for 20 min, +4 °C). The supernatant was discarded and the pellets were washed with 20% TCA followed by three ethanol–ethylacetate (1:1) (v/v) washes. The pellets were then solubilized in 1 ml of 6 M guanidine hydrochloride and 20 mM potassium dihydrogen phosphate (pH 2.3). The carbonyl content was determined by the absorbance measurement at 380 nm and an absorption coefficient of *ε*=22,000 M^−1^cm^−1^.

### Malondialdehyde (MDA)

Plasma MDA levels were assayed with spectrophotometric methods [21]. Lipid peroxidation was quantified by measuring the formation of thiobarbituric acid reactive substances (MDA-TBA). Briefly, 150 μl plasma were added to 25 μl 0,2% BHT and 600 μl 15% aqueous. The mixture was centrifuged at 4000*g* for 15 min at 4 °C. 300 μl of the deproteinized supernatant was transferred in a 2 ml tube and added with 600 μl of TBA (0.375% in 0.25 M HCl). Samples were then heated at 100 °C for 15 min in boiling water. After cooling, sample absorbance were determined spectrophotometrically at 535 nm and compared to standard MDA (1,1,3,3-tetramethoxypropane) solutions.

### Creatine kinase (CK)

Plasma CK activity was determined spectrophotometrically^,^ according to manufactory recommendations, by a manual procedure using a commercial test kit (Greiner Diagnostic GmbH, Bahlingen-Gremany). Briefly, 50 μl plasma were incubated in Hexokinase–Glucose 6 Phosphate–G6P Dehydrogenase buffer for 3 min and then NADPH production was followed at 340 nm for further 3 min.

### Isolation of PBMCs and western blot analysis

Humans PBMCs were purified from whole blood by Ficoll gradient (Sigma-Aldrich, Milan, Italy). The cells were then lysed in lyses buffer (RIPA) and their protein content was determined using the BCA assay (Sigma-Aldrich). Proteins were electrophoresed on an SDS-PAGE and transferred onto a PVDF membrane (Amersham Biosciences, Milan, Italy). The following antibodies were used: Hsp27 (Santa Cruz Biotechnology, CA, USA); β-actin (1: 3000; Sigma-Aldrich); Hsp70 (1: 1000; Stressgen, Florence, Italy). All immunoblots were visualized with horseradish peroxidase-conjugated secondary antibody followed by detection with enhanced chemiluminescence (Amersham Biosciences). Bands were quantified by Image J software. The expression of β-actin was used as a normalizing control.

### Comet assay

To evaluate DNA damage induction in PBMCs, alkaline (pH>13) Comet assay was performed on PBMCs samples taken before, immediately after and 1 h after the graded maximal exercise test. Following the Tice–Vasquez procedure, adapted from the N.P. Singh protocol [22], at the end of treatment, cells were centrifuged for 10 min at 1100 rpm and the pellet re-suspended in PBS. A freshly prepared suspension of cells in 0.75% low melting point agarose (LMA Sigma Chemicals) dissolved in PBS w/o Ca^++^ and Mg^++^ was cast onto microscope slides precoated with 0.5% normal melting point agarose (NMA Sigma Chemicals). The cells were then lysed for 1 h at 4 °C in a lysis buffer consisting of 2.5 M NaCl, 100 mM EDTA, 1% Triton X-100, 10% DMSO, 10 mM Tris, pH 10. After the lysis, DNA was allowed to unwind for 40 min in electrophoretic solution consisting of 300 mM NaOH, 1 mM EDTA, pH>13. Electrophoresis was conducted at 4 °C for 30 min at electric field strength 0.73 V/cm (30 mA). The slides were then neutralized with 0.4 M Tris, pH 7.5, fixed with methanol and stored at RT. The slides were stained with 100 µl Ethidium Bromide 1× and then examined at 20× magnification in an Eclipse fluorescence microscope (Nikon, Tokyo, Japan) attached to a COHU 4910 video camera (Cohu, Inc., San Diego, CA, USA) and connected to a personal computer-based image analysis system, Komet 5.5 Image Analysis System (Kinetic Imaging Ltd, Liverpool, UK). Fifty images were randomly selected from each sample and the comet tail DNA was measured. Two parallel tests with aliquots of the same sample of cells were performed for a total of 100 cells. Each experiment was repeated two times.

Percentage of DNA in the tail (% tail DNA) was analyzed. It is positively correlated with the level of DNA breakage or/and alkali labile sites in the cell. The mean value of the % tail DNA in a particular sample was taken as an index of DNA damage in this sample. Statistical analysis of the Comet data was performed by calculation of median of each experimental point and then analyzing the differences between the means of the median values clustered by treatment.

### Apoptosis analysis

Apoptosis induction was evaluated at single cell level through TUNEL assay on PBMCs samples taken before, 1 h and 24 h after the GXT. Lymphocytes were centrifuged, rinsed twice with PBS and fixed with paraformaldehyde (4% in PBS) at 4 °C for 1 h. After incubation, cells were re-suspended in 500 μl PBS, gently dropped on a clean dry slide coated with poly-l-lysine (BD Biosciences) and air dried. After rinsed with PBS, cells were incubate in permeabilization solution (0.1% Triton X-100 and 0.1% sodium citrate) freshly prepared for 2 min on ice. Cell apoptosis was evaluated by terminal deoxynucleotidyl transferase (TdT)-mediated dUTP-nick end labeling (TUNEL) using “*In situ* cell detection Kit, Fluorescein” (Roche Applied Sciences, Germany). This assay is based on the identification of DNA strand breaks, generated during apoptosis, by labeling free 3′-OH termini with the addition of fluorescein dUTP at strand breaks by terminal deoxynucleotidyl transferase (TdT). Cells were labeled with fluorescein dUTP for 1hr at 37 °C in a humidified chamber in the dark. After, slides were washed 3 times with PBS and mounted in glycerol 50% PBS for the analysis under a fluorescence microscope (Olympus BX41). Apoptosis frequency was evaluated by scoring the number of TUNEL positive nuclei on at least 1000 cells analyzed. Mean and SEM were calculated from three separate cell samples, and all the experiments were performed in triplicate.

### Statistical analyses

All statistical analyses were performed using IBM SPSS Statistics 18 (IBM Corporation). After testing whether data were normally distributed (*Shapiro–Wilk test*), an analysis of variance (ANOVA) with repeated measures for time (pre-training and 12 weeks) and group (trained and control) was performed. In the case of non-homogeneity of variances revealed by the Mauchly's sphericity test (*p*<0.05), the *Greenhouse*–*Geisser* correction was used to assess significant main effects. Where significant main effects were observed, *Bonferroni's* post hoc correction (*p*<0.05) was used to aid interpretation of these interactions. The delta percentage was calculated through the standard formula: Change (%)=[(post test score−pre test score)/pre test score]×100. The statistical power for the main outcome (GSH response) was higher than 80% and it was around 80% also for the secondary outcomes (MDA and protein carbonyls).

## Results

### Redox homeostasis, oxidative damage and creatine kinase release

**Fig. 1:**
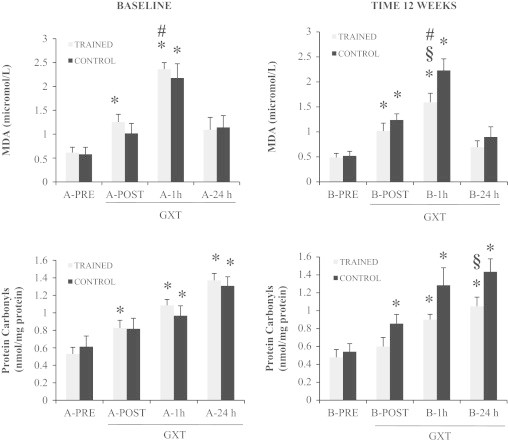
Effect of EMRT and GXT on plasma MDA and protein carbonyls levels. The plasma malondialdehyde (MDA) and protein carbonyls content were measured before (subset A) and after (subset B) the 12 weeks experimental time. Both subsets underwent a graded maximal exercise stress test (GXT). Gray bars: subjects performing the EMRT protocol. Black bars: control subjects. Data are presented as mean±SE; *n*=8. Significant statistical changes: *p<0.05 *vs*. PRE samples; ^§^*p*<0.05 Trained *vs.* Control samples; ^#^*p*<0.05 EMRT *vs.* Baseline.

**Fig. 2:**
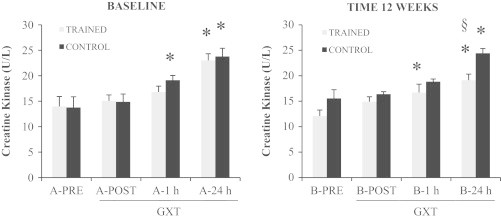
Effect of EMRT and GXT on plasma creatine kinase levels. The plasma creatine kinase (CK) levels were measured before (subset A) and after (subset B) the 12 weeks experimental time. Both subsets underwent a graded maximal exercise stress test (GXT). Gray bars: subjects performing the EMRT protocol. Black bars: control subjects. Data are presented as mean±SE; *n*=8. Significant statistical changes: *p<0.05 *vs*. PRE samples; ^§^*p*<0.05 Trained *vs.* Control samples.

The PRE-GXT value of antioxidant status and cellular damage parameters were comparable between groups (PRE samples, subsets A&B). Indeed their value appeared to be unrelated to training, since we observed similar values also between groups (B-PRE control *vs.* trained) (Figs. 1 and 2 and Table 2). Subjects showed significant responses to the GXT in all groups (Figs. 1 and 2 and Table 2). Actually, a similar trend was observed for all biochemical parameters at baseline (subset A) and after 12 weeks (subset B) (Figs. 1 and 2 and Table 2). Significant group×time differences were found both for oxidized glutathione and for glutathione ratio (GSH/GSSG) (*p*<0.05). After 12 weeks, immediately and 1 h after GXT (B-POST and B-1 h) a lower level of oxidized glutathione was found between groups (B-POST control *vs.* B-POST trained, *p*<0.05) (Table 2). Consequently in B-POST trained group, the GSH/GSSG ratio was higher (*p*<0.05) compared with the other groups (Table 2).

As far as MDA, protein carbonyls and creatine kinase (CK) levels are concerned, we observed significant differences in relation to GXT, in all groups (Figs. 1 and 2). MDA increased immediately after GXT (A&B POST, *p*<0.01), reaching the maximum after 1 h (*p*<0.01). The response to GXT was similar after 12 weeks, but the increases were significantly lower within groups (B-1 hour control *vs.* B-1 h trained, *p*<0.05). Protein carbonyls showed a very similar behavior, being significantly group x time affected by GXT but with a lower value after training (B-24 h control *vs.* B-24 h trained *p*<0.05). In our samples, creatine kinase reached its maximum at 24 h after GXT similarly in control and trained group, but in B-24 h trained, CK level was significantly less pronounced between groups (*p*<0.01; Fig. 2).

### Analysis of Hsp70 and Hsp27 content in PBMCs

**Fig. 3:**
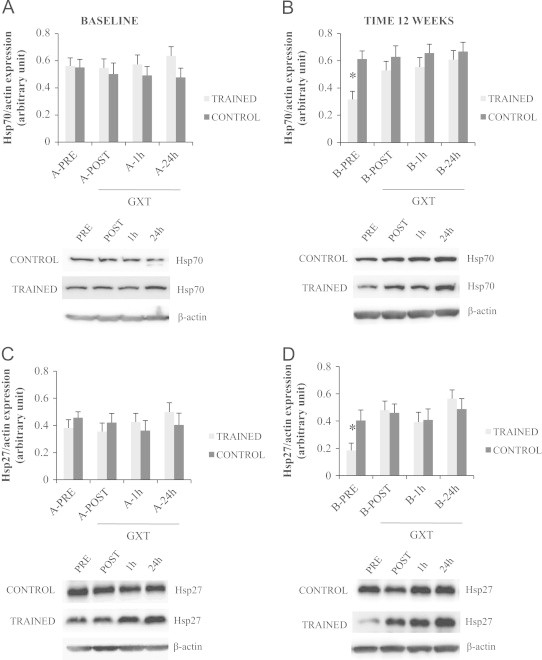
Effect of EMRT and GXT on Hsp27 and Hsp70 levels. The heat shock proteins 27 and 70 content were measured by Western blot analysis in PBMCs before (subset A) and after (subset B) the 12 weeks experimental time. Both subsets underwent a graded maximal exercise stress test (GXT). Gray bars: subjects performing the EMRT protocol. Black bars: control subjects. Protein expression was measured as the ratio between the optical density (OD) of marker protein and the OD of β-actin. In each panel, images show immunoblot results from the same representative subject, while the histograms represent the mean±SE; *n*=8. *p<0.05 *vs.* each other experimental point between groups and within the group.

As we have shown in our previous report [16], Hsp70 and Hsp27 protein levels were reduced subsequent to training compared with baseline (A-PRE trained *vs.* B-PRE trained: Hsp70/β-actin ratio, 0.56±0.09 *vs.* 0.31±0.06, *p*<0.05; Hsp27/β-actin ratio, 0.38±0.06 *vs.* 0.18±0.05, *p*<0.05) (Fig. 3).

Before the training period (subset A), GXT did not induce significant changes among the different time points of sampling. The level of Hsp70 and Hsp27 was not different between groups (trained *vs.* control) and within the groups at any time point analyzed (PRE-POST-1hour-24hours) (*p*>0.05). However, following 12 weeks experimental time (subset B) only trained subjects showed a significant response to GXT for both HSPs starting from immediately after acute exercise (B-PRE trained *vs.* B-POST trained: Hsp70/β-actin ratio, 0.31±0.06 *vs.* 0.53±0.07, *p*<0.05; Hsp27/β-actin ratio, 0.18±0.05 *vs.* 0.48±0.07, *p*<0.05) (Fig. 3).

### DNA damage and apoptosis in PBMCs

Choosing the sample timing was different for Comet and apoptosis. For the Comet assay, the 24 h samples were not taken into consideration because both the immediately and 1 hour after an acute stress samplings are considered the most substantial and accurate in detecting oxidative DNA damages through the Comet assay [23]. On the contrary, for the apoptosis the POST samples should be too early to detect any apoptotic event.

The Comet assay did not reveal any significant difference in DNA damage between controls and trained subjects, neither in relation to the EMRT training, nor with respect the GXT acute exercise (Table 3). The same results were observed for apoptosis rates analysis. No changes were found between groups, both before and after training periods and with respect to GXT (Table 3).

## Discussion

In the present study we were interested to investigate whether a 12 weeks EMRT protocol modulates the capacity of elderly subjects to cope with an acute maximal exercise able to disrupt redox homeostasis and induce oxidative damage. Our results indicated that the regularly trained EMRT older adults have a better antioxidant capacity compared to the age-matched sedentary controls after GXT, a technique known to induce perturbation of the oxidant/antioxidant balance [24,25].

Baseline biochemical and cellular parameters, evaluated before GXT, were not affected by the EMRT protocol. In fact, trained elderly people showed unchanged values of GSH/GSSG ratio compared to control group. Consequently the oxidation target molecules such as the MDA and the protein carbonyls levels as well as the plasma creatine kinase levels were also unaltered in the EMRT group compared to control. Hence, we can confirm and expand our previous results on the absence of induction of oxidative stress by EMRT training in healthy elderly subjects. These results provide further data in support of the beneficial effects of 12 weeks of low frequency, explosive-type moderate resistance training for the elderly, since we previously demonstrated that it was highly effective in eliciting enhanced muscular and functional performance [16].

In this study, we were highly interested to evaluate the effects of the EMRT protocol on the previously indicated systemic markers of oxidative stress, skeletal muscle and oxidative damage after GXT.

The increase in oxidative stress following acute exercise can be reflected by altered antioxidant status [26–28]. In our subjects the evaluation in GSH/GSSG ratio, a parameter routinely performed as a representation of exercise induced oxidative stress and oxidative damage risk [29], showed that GXT induced, as expected significant modification in the GSH system responses. Indeed, redox changes in glutathione are a well-recognized index of altered redox homeostasis and typically, a decrease in reduced glutathione, an increase in oxidized glutathione with no change in total glutathione concentration has been reported following various exercise protocols [29].

In our subjects, GXT temporary affected the redox balance both in sedentary and trained individuals, but in the EMRT group, immediately after GXT a significantly lower GSSG values and concomitantly higher GSH/GSSG ratio were observed. So, even though EMRT practitioners had resting levels of GSH and GSSG comparable to the control subjects, interestingly they responded more effectively to acute oxidative stress than the age-matched sedentary controls.

Previous studies have already shown that physical exercise may induce adaptations that increase resistance to oxidative damage through the up regulation of antioxidant defenses therefore limiting the formation of free radicals in the mitochondria of skeletal muscle [30].

Although we did not measure the activity of antioxidant enzymes, data from other studies have shown that exercise training increases expression of antioxidant enzymes including glutathione peroxidase (GPx), superoxide dismutase, glutathione reductase (GR), and catalase [31,32]. It has been suggested that GPx and GR may be the most responsive enzymes to exercise-induced oxidative stress [33,34]. Hence, we can speculate that individuals from EMRT group may have increased GPx and GR activity, so they may be able to recycle GSSG rapidly while sedentary individuals with lower GPx and GR activity may be more susceptible to oxidative damage resulting from GXT.

An acute, intense muscular effort may induce micro-lesions in active muscle and plasma CK concentration is the most sensitive indirect marker of muscle damage [35]. In EMRT group, the amount of CK was decreased compared to the control group following the GXT. This phenomenon was also paralleled by reduced oxidative damage as assessed through MDA and protein carbonyls levels.

These results show that under basal condition, the moderate intensity and low frequency of the training probably permits enough recovery and did not induce excessive oxidative stress. The differences, between the two groups in oxidative stress markers became readily evident in response to an intense physical challenge, and our findings demonstrate that elderly subjects practicing EMRT seem to better preserve their cellular reducing power after an acute bout of exercise and in doing so they suffer less cellular damage than the resting counterparts.

At cellular level, we evaluated in PBMCs the expression of Hsp70 and Hsp27, two of the most strongly inducible chaperones in response to exercise [36]. As we have already reported [16], the basal expression of both HSPs in the trained group at rest was significantly diminished compared with untrained persons, as if the organism became accustomed to certain repeated exercise-stressor during the training period. After GXT, we observed a significant increase of Hsp70 and Hsp27 only in the trained group, where subjects showed the lower resting values because of the EMRT. We assume that in untrained group, the higher basal levels of both HSPs were sufficient to cope with the physiological stress induced by the acute exercise. Indeed, it was previously reported that when optimal HSPs expression is reached, no further induction of these proteins is observed in response to stress [37,38]. This finding seems to be of high interest since it confirmed that elderly people could benefit from regular exercise maintaining also the ability to modulate HSPs levels in response to positive (EMRT) or negative (GXT) stressors.

Concerning the DNA damage and lymphocyte cell death, in our study there were no differences between controls and trained subjects with respect to basal values of apoptosis rates and DNA damage, showing the homogeneity of the groups and the absence of sampling bias. This pattern was conserved even after the training period for both the parameters observed before and after GXT. This phenomenon could be ascribed to the fact that, in our subjects, the bout of physical exercise was not long enough to represent a stimulus for the activation of some apoptotic pathways as well as to cause any significant DNA damage detectable by Comet assay. This hypothesis is supported by literature data showing that DNA damage and apoptotic response caused by exercise depends also on the type/intensity/duration of the physical effort [39,40].

In conclusion, we showed for the first time that EMRT power training, an effective workout that elicits a significant enhancement in muscular strength and power, and functional performance, is also able to improve the general adaptive response to oxidative stress induced by intense acute exercise, therefore may be proposed as an effective intervention for improving the overall health of the older people.

## Figures and Tables

**Table 1:** Baseline participant characteristics.

	**CONTROL BASELINE**	**TRAINED BASELINE**
**Subjects**	***n*****=8**	***n*****=8**
**Age (years)**	74±2	71±2
**Weight (kg)**	73±3	71±4
**BMI (kg/m**^**2**^**)**	26±1	26±2

**Physical activity level #**	18±2	21±2
**Household score**	13±1	15±2
**Sports score**	3±1	3±1
**Leisure score**	3±1	4±1


**After 12 weeks protocol**	CONTROL	TRAINED

**Δ Strength (%)**		
**Leg extension**	−0.3±1.5	16.4±1.5*§
**Leg curl**	−1.7±1.6	15.3±1.1*§
**Low row**	−1±0.6	14.6±1.2*§
**Chest press**	0.6±0.7	19.6±1.9*§

**Δ Power (%)**		
**Leg extension**	−0.9±1.9	35.9±5.2*§
**Leg curl**	−2.6±3.1	27.9±5.4*§
**Low row**	−5.7±4.8	28.2±4.5*§
**Chest press**	−5.6±3.4	33.8±3.4*§
**CMJ**	−2±2.8	17.5±4.3*§

*# Modified Backle questionnaire*. All values represent mean±standard error. BMI, body mass index. CMJ, Counter movement jump. Differences between groups (CONTROL *vs*. TRAINED GROUP) were not significant.

§ *p*<0.05 *vs.* BASAL sample.

⁎ *p*<0.05 *vs.* CTRL sample.

**Table 2:** Blood glutathione homeostasis.

**SAMPLES**	**BASELINE**				**TIME 12 WEEKS**			
		**GXT**				**GXT**		
	**A-PRE**	**A-POST**	**A-1 h**	**A-24 h**	**B-PRE**	**B-POST**	**B-1 h**	**B-24 h**
**CONTROL (*****n*****=8)**								
**tGSH (10**^**−5**^**M)**	83.57±8.00	82.57±8.02	77.66±8.37	78.95±7.87	85.58±7.49	79.64±7.71	81.55±7.02	81.74±6.97
**GSSG (10**^**−5**^**M)**	10.64±0.58	16.74±0.67⁎	14.72±0.67⁎	12.47±0.57⁎	11.63±0.76	17.91±0.96⁎§	15.41±0.83⁎	11.68±1.04
**GSH/GSSG**	7.86±4.50	4.93±7.17⁎	5.28±6.70⁎	6.33±5.59	7.36±4.80	4.45±7.95⁎§	5.29±6.68⁎	7.01±5.05

**TRAINED (n=8)**								
**tGSH (10**^**−5**^**M)**	80.03±5.59	73.27±4.80	75.23±4.28	77.25±4.19	85.66±5.14	85.12±4.67	81.06±5.06	79.35±5.05
**GSSG (10**^**−5**^**M)**	11.21±0.66	16.67±1.03⁎	14.40±1.26⁎	12.97±0.76	11.36±0.74	14.76±0.72⁎§	14.00±0.90⁎	12.61±0.58
**GSH/GSSG**	7.23±4.89	4.40±8.04⁎	5.22±6.76	5.95±5.94	7.54±4.69	5.77±6.13§	5.79±6.11	6.29±5.62

The total glutathione (tGSH) and oxidized (GSSG) glutathione content and GSH/GSSG ratio were measured before (subset A) and after 12 weeks of experimental time (subset B). Samples were collected before (PRE), immediately after (POST), 1 h and 24 h after the GXT. Data are presented as mean±SE.

⁎ Significant statistical change: *p*<0.05 *vs.* PRE samples.

§ Significant statistical change: *p*<0.05 Trained *vs.* Control samples.

**Table 3:** Changes in DNA damage (Comet assay) and apoptosis rate.

**SAMPLES**	**BASELINE**				**TIME 12 WEEKS**			
		**GXT**				**GXT**		
	**A-PRE**	**A-POST**	**A-1 hour**	**A-24 hours**	**B-PRE**	**B-POST**	**B-1 hour**	**B-24 hours**
**CONTROL(*****n*****=8)**								
**DNA in tails (%)**	5.73±0.53	5.75±0.53	5.67±0.36	n.d.	7.65±1.46	6.42±0.74	7.75±1.11	n.d.
**Apoptosis rate (% apopotic nuclei)**	1.10±0.51	n.d.	0.85±0.48	0.84±0.49	1.37±0.47	n.d.	0.83±0.43	0.94±0.58

**TRAINED (*****n*****=8)**								
**DNA in tails (%)**	7.24±0.69	5.76±0.62	4.76±0.62	n.d.	6.26±0.62	6.74±0.78	6.25±0.80	n.d.
**Apoptosis rate (% apopotic nuclei)**	0.96±0.31	n.d.	0.82±0.14	0.97±0.12	1.41±0.60	n.d.	0.91±0.10	0.77±0.09

DNA damage (Comet assay) and apoptosis rate (% apopotic nuclei) were measured before (subset A) and after 12 weeks of experimental time (subset B). Samples were collected before (PRE), immediately after (POST), 1 h and 24 h after the GXT. n.d., not determined. Data are presented as mean±SE.
